# Genomic Copy Number Variation Affecting Genes Involved in the Cell Cycle Pathway: Implications for Somatic Mosaicism

**DOI:** 10.1155/2015/757680

**Published:** 2015-09-01

**Authors:** Ivan Y. Iourov, Svetlana G. Vorsanova, Maria A. Zelenova, Sergei A. Korostelev, Yuri B. Yurov

**Affiliations:** ^1^Mental Health Research Center, Moscow 117152, Russia; ^2^Separated Structural Unit “Clinical Research Institute of Pediatrics”, Pirogov Russian National Research Medical University, Ministry of Health of Russian Federation, Moscow 125412, Russia; ^3^Department of Medical Genetics, Russian Medical Academy of Postgraduate Education, Moscow 123995, Russia; ^4^I.M. Sechenov First Moscow Medical University, Moscow 119991, Russia

## Abstract

Somatic genome variations (mosaicism) seem to represent a common mechanism for human intercellular/interindividual diversity in health and disease. However, origins and mechanisms of somatic mosaicism remain a matter of conjecture. Recently, it has been hypothesized that zygotic genomic variation naturally occurring in humans is likely to predispose to nonheritable genetic changes (aneuploidy) acquired during the lifetime through affecting cell cycle regulation, genome stability maintenance, and related pathways. Here, we have evaluated genomic copy number variation (CNV) in genes implicated in the cell cycle pathway (according to Kyoto Encyclopedia of Genes and Genomes/KEGG) within a cohort of patients with intellectual disability, autism, and/or epilepsy, in which the phenotype was not associated with genomic rearrangements altering this pathway. Benign CNVs affecting 20 genes of the cell cycle pathway were detected in 161 out of 255 patients (71.6%). Among them, 62 individuals exhibited >2 CNVs affecting the cell cycle pathway. Taking into account the number of individuals demonstrating CNV of these genes, a support for this hypothesis appears to be presented. Accordingly, we speculate that further studies of CNV burden across the genes implicated in related pathways might clarify whether zygotic genomic variation generates somatic mosaicism in health and disease.

## 1. Introduction

Somatic mosaicism (somatic genome variations) has long been considered as a source for human genomic diversity and pathology [1–3]. However, causes and consequences of postzygotic genomic variation (i.e., loss/gain of chromosomes in a cell or aneuploidy) remain largely unknown. The latter is probably the reason for mosaicism underappreciation in current genomic research [2–4]. To date, somatic genome variations have been observed in almost all healthy human tissues [3–6]. Interestingly, somatic genetic changes more commonly manifest as aneuploidy [2–6]. Furthermore, it has been repeatedly shown that somatic aneuploidy is likely to be a mechanism for a variety of diseases [7–13]. Assessing causes and consequences of somatic genome variations, a hypothesis, suggesting genomic changes to be acquired during the lifetime because of natural zygotic genomic variation, has been proposed [14]. Since common types of somatic mosaicism (mainly postzygotic aneuploidy) are likely to result from alterations in cell division (mitotic) regulation and genome maintenance pathways [4, 13–15], it has been hypothesized that zygotic (heritable and sporadic) genomic variation across genes implicated in pathways related to cell cycle regulation is the most likely cause of intercellular genome diversification [14]. Consequently, a simple analysis of genomic copy number variation (CNV) in genes implicated in these pathways is able to answer the question whether this hypothesis is worth further testing.

In the present study, we have performed an analysis of genomic CNV affecting genes implicated in the cell cycle pathway (hsa04110 from the Kyoto Encyclopedia of Genes and Genomes or KEGG) by high-resolution molecular karyotyping (SNP-microarray analysis) in a cohort of 225 children with intellectual disability, autism, epilepsy, and/or congenital malformations. Genomes of these individuals were addressed inasmuch as their phenotypes had resulted from genomic rearrangements (chromosome abnormalities), which had not affected genes implicated in this specific pathway.

## 2. Materials and Methods

### 2.1. Study Subjects

Genomes of 225 children with intellectual disability, autism, epilepsy, and/or congenital malformations from a cohort (~2500 patients) that has been partially described in a previous study [16] were analyzed. These individuals were selected according to results of molecular karyotyping, which showed occurrence of genomic rearrangements (chromosome abnormalities) relevant to the phenotypes without affecting genes implicated in the cell cycle pathway (hsa04110 from KEGG). Patients' ages varied between 1 month and 18 years. Written informed parental consent was obtained for each individual.

### 2.2. CNV Analysis

Genomic CNVs were analyzed using CytoScan HD Arrays (Affymetrix, Santa Clara, CA) consisting of approximately 2.7 million markers for CNV evaluation and approximately 750,000 SNPs. CNVs were addressed by the Affymetrix Chromosome Analysis Suite (ChAS) software (ChAS analysis files for CytoScan HD Array version NA32.3). Genomic localization and gene content of detected CNVs were defined using NCBI Build GRCh37/hg19 reference sequence. The procedures have been previously described in detail [17–24].

### 2.3. Data Analysis

Data analysis was performed using a bioinformatic workflow described recently [25]. Data on individual CNV profiling was analyzed against all the genes indicated to be involved in the cell cycle pathway indexed in KEGG (http://www.genome.jp/dbget-bin/www_bget?pathway+hsa04110). Inclusion criteria were referred to either a CNV affecting whole gene or an intragenic exonic copy number change. Causative CNVs (defined by a protocol of CNV prioritization [25]), submicroscopic genomic rearrangements, or larger chromosome abnormalities affecting these genes were all excluded from the analysis.

## 3. Results and Discussion

**Figure 1:**
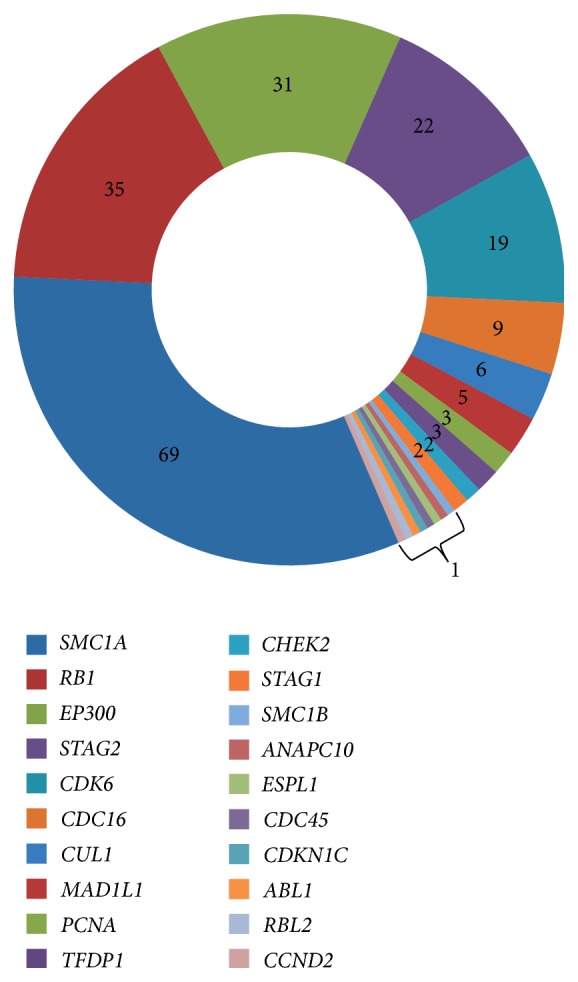
Distribution of genomic CNVs (numbers correspond to amount of individuals demonstrating CNV affecting a gene) across genes implicated in the cell cycle pathway (hsa04110).

CNVs affecting genes implicated in the cell cycle pathway according to the KEGG (http://www.genome.jp/dbget-bin/www_bget?pathway+hsa04110) were found in 161 patients (71.6%). Twenty genes were affected in a variable manner (Figure 1). In total, 214 CNVs have been detected. Recurrent CNVs affected* SMC1A*,* RB1, CDC16*, and* CUL1* (deletions),* STAG2* (duplications), and* CDK6* and eighth exon of* EP300 *(four copies). It is to note that these genes were also affected by nonrecurrent CNVs. Deletions (one copy) have been observed in 70.6% (151 CNVs), duplications (three copies) in 16.8% (36 CNVs), and four copies in 12.6% (27 CNVs). In* MAD1L1*, deletions and a copy number increase (four copies) were detected. In* PCNA*,* CHEK2*,* STAG1*,* SMC1B*,* CDC45*, and* ABL1* deletions were observed. In* TFDP1*,* ESPL1*, and* CDKN1C *duplications were found. In* ANAPC10*,* RBL2*, and* CCND2* other types of copy number increase (four copies) were detected.

Recurrent CNVs (apart from four copies of* CDK6* and eighth exon of* EP300*) were all colocalized with genomic variations indexed in the Database of Genomic Variants of The Centre for Applied Genomics (TCAG) hosted databases at The Hospital for Sick Children (SickKids) (http://dgv.tcag.ca/dgv/app/home), whereas nonrecurrent CNVs were not found to correspond to genomic variations from the reference databases of benign genomic changes. These results suggest that a number of detected CNVs are common in general population.

**Figure 2:**
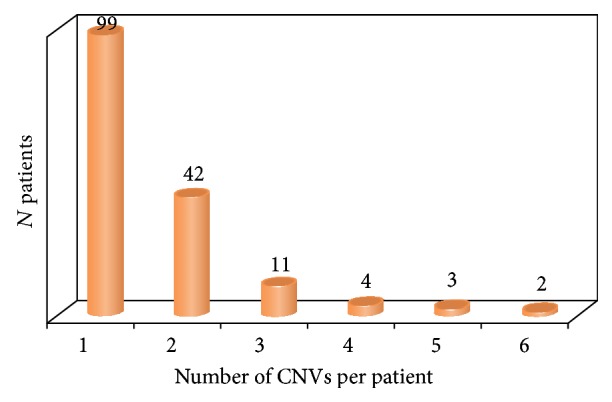
Individual incidence of CNV affecting genes implicated in the cell cycle pathway.

Single CNVs affecting a gene implicated in the cell cycle pathway were found in 99 individuals. In the remainder, the incidence of the CNVs was as shown in Figure 2. Patterns of individual incidence of the CNV affecting genes implicated in the cell cycle pathway allowed us a suggestion that a kind of CNV burden across the genes implicated in cell cycle pathways is likely to exist in at least 38.5% of individuals demonstrating genomic variations altering related pathways. Thus, discussions concerning a specific “cell cycle” CNV burden do not appear too speculative.

There is a line of evidence that somatic mosaicism is common in humans. Although somatic genome variations manifesting as structural chromosomal or genomic rearrangements are occasionally reported in unaffected population [3, 10, 26–28], numerical chromosome abnormalities (aneuploidy and more rarely polyploidy including tissue-specific chromosomal mosaicism) [7, 12–14, 29–32] and small supernumerary marker chromosomes [33] are a common cause of somatic mosaicism. In addition, our cohort has been previously analyzed in terms of stochastic somatic chromosomal mosaicism and almost all individuals demonstrated low-level mosaic aneuploidy [3, 25, 34, 35]. Moreover, human postmitotic tissues (i.e., adult human brain) demonstrate intercellular genomic variation essentially manifesting as low-level aneuploidy [32, 36–40]. Together, this suggests that a genomic background (i.e., CNV burden) for somatic genome diversification generated by alterations in cell cycle (genome stability) regulation pathways is likely to exist. The latter has been partially confirmed by studies of somatic genome variations mediating neurodegeneration resulting from alterations in cell cycle regulation and genome stability maintenance pathways [41–43]. Finally, numerous monogenic, chromosomal, and complex diseases are hypothesized to be associated with somatic mosaicism concomitant with failure of safeguarding genome and cell cycle machineries or genomic variations in related genes* per se* [44–51]. In the light of this study, it seems attractive to link presumably benign zygotic (sporadic or inherited) genomic variations slightly changing cell cycle pathway and somatic mosaicism. Alternatively, a heavier “cell cycle” CNV burden can be designated as a mechanism for a broad spectrum of diseases associated with somatic genome variations manifesting later in life [52, 53].

Somatic genome variations are considered to have prenatal origin. Developmental chromosome and genome instability hallmarks human prenatal development at cellular and tissular levels [54–60]. The following ontogenetic stages are also associated with changes of somatic cellular genomes. For instance, aging has long been documented to be associated with accumulation of sporadic somatic mutations, which were hypothesized to be produced either by exhaustion of mitotic and cell death machineries or by genomic variations affecting genes implicated in these pathways [38, 60–64]. Accordingly, ontogenetic genomic variation has been also attributed to these cellular pathways [65, 66]. Similarly, addressing pathological aging of postmitotic tissues, it has been shown that these pathways are more likely to be inheritably altered rather than experience adverse changes during the lifespan [46, 67–69]. Nevertheless, environmental effects triggering accumulation of somatic mutations mediated by cell cycle errors represent an important contribution to healthy/unhealthy aging and a variety of aging and late onset diseases [70–74]. Consequently, our data supports the hypothesis about germline origins of genomic variations affecting genes implicated in cell cycle pathways that do predispose to somatic genome variations mediated by genetic-environmental interactions. In this context, one can propose that a specific “cell cycle” CNV burden would be a key element in the pathogenic cascade initiated by constitutional (nonmosaic) genomic variation and culminated by somatic mosaicism.

CNV burden is a clinically valuable parameter that is important for assessing disease mechanisms and phenotypic significance of genomic variations [75–78]. However, this phenomenon has not been evaluated in cases of somatic mosaicism [25, 79]. An attempt at filling this gap by our preliminary data is pertinent inasmuch as the lack of an integral view on interaction between heritable/sporadic germline and somatic genome variations produces numerous discrepancies between empirical data acquired through single-cell analysis and generalized data on genome variability brought by “classical” strategies targeting DNA fractions isolated from large cell populations [79–81]. In this instance, mechanisms underlying intercellular genomic heterogeneity are likely to be referred to a predisposition of cellular genome to change. This suggests uncovering the basis of cellular genome susceptibility to vary throughout ontogeny to be of fundamental importance for current genomics and molecular genetic diagnosis.

Here, we have used KEGG for addressing contribution of CNVs to possible susceptibility to chromosome instability and to origin of somatic mosaicism.* In silico* analysis of CNV data has been considered contributive to definition of genetic mechanisms on the basis of molecular cytogenetic data [25]. Recently, KEGG-based selection/filtering of genes implicated in “pathways of interest” was found to be efficient for elucidating the molecular mechanisms of processes such as genome/chromosome instability and carcinogenesis involving genes found to be affected by CNVs in the present study [82–84]. Consequently, we concluded that gene ontology analysis of a single pathway in context of natural (presumably benign) CNVs is able to show whether further testing of the aforementioned hypothesis [52] would be productive.

The present data demonstrates that there do exist more-or-less common recurrent CNVs affecting 5 genes (*SMC1A, RB1, EP300, STAG2, *and* CDK6*) and rare but recurrent CNVs affecting 5 genes (*CDC16, CUL1, MAD1L1, PCNA, *and* TFDP1*) implicated in the cell cycle pathway (Figure 1). One can notice that detected CNVs are able to produce susceptibility to cancer mediated by chromosome/genome instability [85–87], which is rather predictable in the light of the involvement in the cell cycle pathway. In addition, a number of these genes are mutated in hereditary diseases. On the other hand, following guidelines on determination of CNV pathogenic value [88, 89] strongly evidences that these genomic changes are likely either to be benign or to produce a susceptibility to common diseases or traits. The latter can be considered mechanisms for increasing background levels of somatic (stochastic) mutations. Among genes implicated in the cell cycle pathway,* SMC1A* was most commonly involved in CNVs. This gene mutated in Cornelia de Lange syndrome and colorectal cancers [90] is involved in G2/M arrest in humans [91]. The second gene is* RB1* (retinoblastoma tumor-suppressor gene) representing a well-known inhibitor of cell cycle progression, alterations to which can cause aneuploidization and other processes initiating genome instability in cancers [92, 93]. One can speculate that these recurrent CNVs are able to render cells susceptible to chromosome instability.* EP300* is mutated in a small proportion of Rubinstein-Taybi syndrome cases [94] and in cancers exhibiting instable genomes, which can be a result of alterations to chromatin-remodeling [95]. Although inactivating point mutations in* STAG2 *are not likely to be directly related to aneuploidy [96], more recent studies have shown that frequent sequence variations are inversely related to chromosomal copy number changes [97].* CDK6 *mutations causing clinical conditions and several cancers are involved in processes related to aneuploidization [98, 99]. Finally,* CDC16, CUL1, MAD1L1, PCNA, *and* TFDP1* were all found to be integrated into a network of the cell cycle pathway, which is likely to be responsible for cancer progression [100] and involved in genome/chromosome instability. Thus, evaluating functional consequences of CNVs affecting the aforementioned genes is able to provide a basis for speculations concerning the ability of these apparently benign CNVs to be responsible for susceptibility to chromosome (genome) instability or somatic mosaicism in presumably normal tissues.

Our study provides a preliminary support for a hypothesis suggesting zygotic (sporadic and heritable) genomic variation to form a susceptibility to cellular genome instability or somatic genome variations (mosaicism) through genetic variability affecting genes implicated in cell cycle genome maintenance regulation pathways. Since this hypothesis appears to be valid at least in case of the cell cycle pathway (hsa04110), one may speculate that future studies targeted at evaluating related pathways (i.e., mitotic chromosome segregation, DNA reparation/replication, genome stability maintenance, etc.) are able to clarify whether zygotic genomic variation can generate somatic genome variation in health and disease.

## 4. Conclusion

Our preliminary study has shown that natural CNV affecting genes implicated in the cell cycle pathway is relatively common. It is noteworthy that a significant proportion of individuals with these CNVs carry a kind of CNV burden across genes implicated in the cell cycle pathway. These data provide an experimental support for the hypothesis suggesting natural zygotic genomic variation (heritable and sporadic) predisposing to nonheritable/postzygotic genomic changes (aneuploidy) affecting genes implicated in cell cycle regulation or related pathways acquired during the lifetime. Since an analysis of a single pathway, alterations in which result in somatic mosaicism (aneuploidy), could support the hypothesis, one may assume that increasing the numbers of pathways analyzed in this context would certainly give further insights into origins of somatic mosaicism and determine intrinsic interactions between zygotic and postzygotic genome variation.
